# Myopia Management in Ontario, Canada †

**DOI:** 10.3390/jcm14145132

**Published:** 2025-07-19

**Authors:** Amy H. Y. Chow, Barbara Caffery, Sarah Guthrie, Mira Acs, Angela Di Marco, Stephanie Fromstein, Stephanie Ramdass, Vishakha Thakrar, Shalu Pal, Matthew Zeidenberg, Deborah A. Jones

**Affiliations:** 1 School of Optometry and Vision Science, University of Waterloo, 200 Columbia Street West, Waterloo, ON N2L 3G1, Canada; sarah.guthrie@uwaterloo.ca (S.G.); mzeidenberg@uwaterloo.ca (M.Z.); debbie.jones@uwaterloo.ca (D.A.J.); 2 Centre for Eye & Vision Research, Hong Kong; 3 Toronto Eye Care, Toronto, ON M4W 1A5, Canada; 4 Dr. Shalu Pal & Associates, Toronto, ON M5S 2V1, Canada; 5 Eyeacademy.ca, 800 Southdown Rd Unit A2, Mississauga, ON L5J 2Y4, Canada; 6 Vaughan Vision Centre, Toronto, ON L4K 0H2, Canada

**Keywords:** myopia management, pre-myopia, myopia, myopia control, retrospective review

## Abstract

**Objectives:** To determine how optometrists in Canada manage their pediatric myopia patients and to assess whether this has changed over time. **Methods:** In a retrospective chart review, records for children aged 6–10 years who had an eye exam between 2017 to 2021 were reviewed. Children were grouped by presenting refraction (myopes ≤ −0.50 D or pre-myopes ≤ +0.75 D). Up to five unique patients were selected for each age (6, 7, 8, 9, and 10) and initial visit year (2017 to 2021) for each group (myopes and pre-myopes), for a maximum of 250 files per practice. Demographic information, refraction, and recommended interventions were recorded. Logistic regression was used to model the likelihood of being prescribed a myopia control intervention based on patient and optometrist characteristics. **Results:** A total of 2905 patients (*n* = 1467 (50%) female) from 15 practices across Ontario, Canada, were included, accounting for 8546 visits. Optometrists predominantly prescribed single-vision spectacle correction as a first-line intervention for myopic children, although this declined from 98.2% in 2017 to 56.7% in 2023. The use of myopia control modalities increased from 1.8% to 43.3% over this same period. Optometrists began recommending myopia control at lower myopic refractive errors over time (−2.63 DS in 2017 vs and −1.49 DS in 2020). Myopia control spectacles were the most commonly prescribed intervention, despite the observation that optometrists are not hesitant to fit contact lenses in younger children. Optometrists who had been in practice longer were more likely to prescribe older forms of myopia control (e.g., bifocals/progressives) than more recent graduates. **Conclusions:** While single-vision spectacle correction remains a primary approach for initial myopia management in Ontario, Canada, optometrists increasingly recommend myopia control and are initiating interventions earlier.

## 1. Introduction

Myopia has become a significant public health concern, with its global prevalence increasing at an alarming rate, especially among children and adolescents [1]. Early-onset myopia is particularly concerning, as children who develop myopia at a younger age tend to experience more rapid progression, increasing their likelihood of developing high myopia by adulthood [2]. Projections from Holden et al. indicate that global myopia prevalence will rise from 30% in 2020 to approximately 40–50% globally by 2050, with 10% of the world’s population having high myopia [3,4]. The impact of myopia extends beyond the need for a vision correction to potential vision impairment. This can lead to diminished quality of life, as well as resultant productivity losses, limitations in education and employment opportunities, and increased dependency among affected individuals [5,6,7].

Healthcare professionals are increasingly recognizing that managing myopia involves more than simply correcting the refractive error. The complications of myopia progression, driven primarily by axial elongation of the eye, include myopic macular degeneration, retinal detachment, glaucoma, and cataracts [8]. These conditions pose a significant threat to long-term vision, potentially resulting in permanent vision loss and a substantial decline in quality of life. For high myopia, the risks of severe vision loss impose additional healthcare challenges, demanding long-term management and treatment of associated complications [9]. The global cost of managing myopia and associated complications is estimated to be in the billions, encompassing direct healthcare expenditures and indirect costs from lost productivity [10]. These escalating economic pressures underscore the urgent need for comprehensive strategies aimed at the prevention, early detection, and management of childhood myopia on a global scale.

There is evidence of a multifactorial etiology for myopia, with environmental factors such as prolonged near work, limited outdoor activities, and genetic predisposition playing pivotal roles in its development and progression [11]. Increasing outdoor time has been shown to be effective in delaying the onset of myopia [12,13,14,15,16,17,18]. Optical approaches (myopia control contact lenses and spectacles) and pharmacological treatments (low-dose atropine) can significantly reduce the rate of progression [19,20,21]. However, despite the strong evidence base, the adoption of myopia control strategies has been limited. A survey of over 3000 eye-care practitioners revealed that, despite increasing awareness of the growing incidence of pediatric myopia and heightened concern, single-vision (SV) spectacles remain the most frequently prescribed refractive correction for myopic children, revealing a significant gap between evidence-based recommendations and clinical practice [20]. The lack of standardized guidelines further complicates myopia management globally, despite the World Council of Optometry’s (WCO) resolution recognizing myopia control as the standard of care [22]. In 2022, the Canadian Association of Optometrists produced a position paper supporting the position of the WCO endorsing evidence-based myopia control as the standard of care for all at-risk patients [23]. A similar endorsement statement was released by Optometry Australia in 2024. Additionally, access to myopia control modalities varies globally. While Canada has access to all evidence-based myopia control options, disparities in available management options persist in other parts of the world.

Retrospective chart reviews offer greater insight into clinical practice patterns compared with practitioner surveys, as analysis can be stratified by age and refractive error. Clinical records also provide longitudinal data, capturing changing management patterns over time. In contrast, practitioner surveys rely on self-reported data, which can be influenced by recollection bias, ultimately distorting findings and reducing their reliability.

This study aimed to examine how practitioners are managing childhood myopia and whether practice prescribing patterns are changing over time, as more myopia control modalities became available in one Canadian province. Myopia control contact lenses were first introduced in 2018, followed by the availability of spectacle myopia control options in 2019. Children 19 years of age and under are eligible for eye exams and subsequent follow-ups through a provincial health program. Optometrists in Ontario can independently prescribe therapeutics (such as atropine), as well as any spectacle or contact lens modalities (including those specifically approved for myopia control or used off-label for myopia control). This analysis will provide insights into evolving myopia management practice patterns and barriers to adopting myopia control as the standard of care. These findings have implications not only for Canada, but also globally to address this growing concern.

## 2. Methods

Optometry practices across Ontario, Canada, were surveyed for their interest in participating in a general chart review research study. Of the 33 interested practices, 15 practices were selected, with a preference for practices who saw more children monthly. Interested practices were sorted into geographic regions across Ontario (Central, East, Southwest, Northeast, Northwest, and Greater Toronto Area) and up to 4 practices could be included per region. The retrospective chart review included patients aged 6–10 years with presenting (with or without cycloplegia) refraction at their first encounter of ≤−0.50 D (myopes) and ≤+0.75 D (pre-myopes) who had comprehensive eye exams from 2017 through 2021 at optometry practices in Ontario. A patient list was generated at each practice for patient age and visit years of interest, and random sampling was used to find patients who met the inclusion criteria. The inclusion and exclusion criteria are detailed in Table 1. A patient’s first visit was defined as a visit within our study period of 2017 to 2021. For a balanced design, up to five unique charts were selected for each age (age 6, 7, 8, 9, and 10) and visit year (2017, 2018, 2019, 2020, and 2021), for each group (myopes and pre-myopes) if inclusion criteria were met (Table 1), for a maximum of 250 unique patient charts.

The chart review captured demographics, refractive data, and refractive management strategy. Data from all subsequent visits for each patient were recorded. Interventions of interest included: no prescription recommended, SV correction (SV spectacles or SV contact lenses (CLs)), bifocal spectacles, progressive spectacles, vision therapy, ortho-keratology (ortho-K), atropine, myopia control spectacles, myopia control CLs, lifestyle changes (increasing outdoor time and reducing screen time), and myopia control discussion. The study protocol was approved by the institutional ethics committee at the University of Waterloo. Study data were collected and managed using REDCap (v10.6.28, Vanderbilt University, Nashville, TN, USA) electronic data capture tools hosted at the University of Waterloo. This study was conducted under a Waiver of Informed Consent. This study was designed to be in conformance with the ethical principles in the Declaration of Helsinki and with the ICH guidelines for Good Clinical Practice.

### Statistical Methods

Statistical analyses were performed using Statistica (version 13, TIBCO Software Inc, Palo Alto, CA, USA) and JASP software (version 0.17.1, Amsterdam, The Netherlands). Both parametric and non-parametric analyses were used depending on the results of Levene’s test for equality of variances. The Kruskal–Wallis test was used to examine whether the prevalence of myopia management changed over time and evaluate whether modalities for first-line intervention differed based on a patient’s age and refractive error or a prescribing optometrist’s year of graduation. The refractive error and age at which myopia control was first prescribed were analyzed with a 2 (grouped by treatment type: SV myopia correction or myopia control management) × 5 (year: 2017 to 2021) analysis of variance. Binomial logistic regression was used to investigate the association between the log odds of being prescribed a myopia control intervention and clinical factors such as patient attributes (patient age at visit, refractive error at visit, and parental myopia) and optometrist attributes (optometrist’s gender, graduation year, and alma mater, grouped by country). Multicollinearity was assessed with the variance inflation factor (VIF). Statistical significance was determined as *p* < 0.05.

## 3. Results

### 3.1. Demographics

A total of 2905 patient charts (*n* = 1467 female) were included in this study, encompassing a total of 8546 visits from 15 practices across Ontario, Canada. Data were extracted consecutively from practices between May 2022 and February 2024. Patient visits spanned January 2017 to February 2024. After patients were sorted by spherical equivalent refractive error at their initial encounter, there were 1160 myopes and 1745 pre-myopes. Of these patient charts, 2077 (70.9%) had multiple documented visits. The focus of this manuscript is on myopic patients (*n* = 1160), as well as any pre-myopic patients who became myopic at subsequent visits (*n* = 193; total *n* = 1353), with demographic details of included patients in Table 2.

### 3.2. Prevalence of Myopia Management over Time

**Figure 1:**
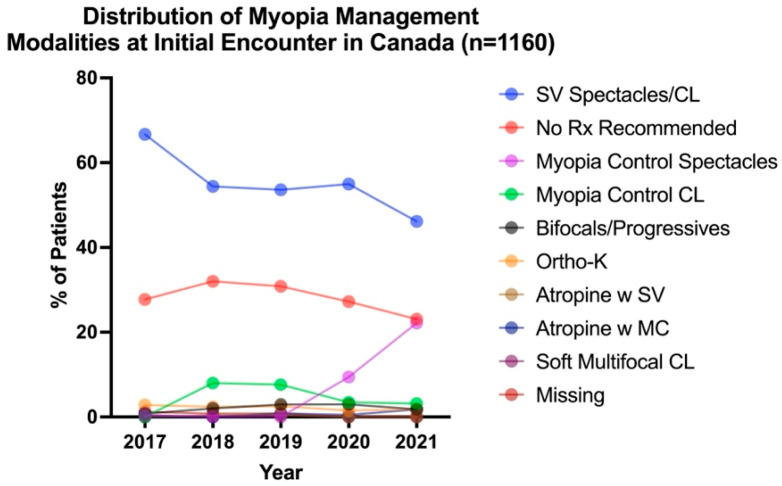
Distribution of myopia management modalities for each patient (*n* = 1160) at their initial encounter from 2017 to 2021.

To examine whether patterns of myopia management had changed over time, the management plan at each patient’s initial visit (*n* = 1160) was determined and represented in Figure 1. The Kruskal–Wallis test revealed a significant effect of time across all prescribed modalities (*H*_4,813_ = 109.05, *p* < 0.001). The most common intervention for myopes (≤−0.50 DS at initial visit) was SV spectacles or SV CL across all years studied. The use of SV devices decreased over time from 66.7% in 2017 to 46.1% by 2021 (Figure 1). The second-most-prevalent form of pediatric myopic management was monitoring with no intervention across all years studied (no change over time, average 28.2%). The prevalence of optometrists prescribing any form of myopia control to their patients increased from 4.4% of optometrists in 2017 to 12.8% in 2018, 14.8% in 2019, 17.9% in 2020, and 30.8% in 2021 (see Supplementary Materials for detailed breakdown). The most prevalent myopia control treatment prescribed was ortho-K in 2017 (2.8%), myopia control CLs in 2018 and 2019 (8.0% and 7.6%, respectively), and myopia control spectacles in 2020 and 2021 (9.4% and 22.2%, respectively). Comparing all years, myopia control spectacles were the most commonly prescribed myopia control device, making up 52.5% of all myopia control modalities in 2020 and 72.1% in 2021. While SV spectacles/CLs were the most common modalities prescribed across all years studied, the uptake of myopia control interventions significantly increased over time.

### 3.3. First-Line Intervention—Patient and Optometrist Factors

**Figure 2:**
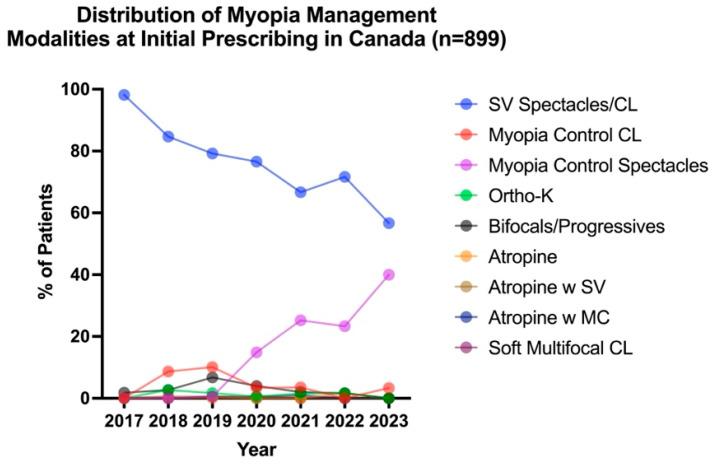
Distribution of first-line intervention modalities across all years reviewed for all myopes (including pre-myopes who became myopic in subsequent visits), categorized by when management was altered (*n* = 899).

To evaluate how first-line interventions changed over time, all myopic patients (*n* = 1353) were analyzed. After excluding myopes who were monitored with no correction, 899 patients were prescribed an intervention between 2017 and 2021, and Figure 2 illustrates the distribution of first-line management modalities, categorized by the year in which the initial prescribing occurred. A Kruskal–Wallis test revealed a significant effect of time on first-line intervention choices for pediatric myopia (*H*_5,892_ = 110.50 *p* < 0.001). Although SV correction remained the most prevalent first-line intervention for pediatric myopia across all years reviewed, it decreased from 98.2% in 2017 to 56.7% in 2023. Nevertheless, the use of myopia control modalities significantly increased from 1.8% in 2017 to 43.3% in 2023. The most common myopia control modality prescribed as first-line intervention was spectacle bifocal/progressives in 2017 (1.8%), myopia control CLs in 2018 and 2019 (8.7% and 10.1%, respectively), and myopia control spectacles in 2020 to 2023 (14.9% in 2020, 25.3% in 2021, 23.3% in 2022, and 40.0% in 2023). Myopia control spectacles were the most commonly prescribed modality in first-line myopia management.

Further analysis evaluated whether factors such as patient age, patient refractive error, and the optometrist’s year of graduation differed across chosen intervention modalities.

#### 3.3.1. Patient Age

A Kruskal–Wallis test revealed no significant effect of patient age on the selection of initial management modalities (*H*_5,892_ = 7.32, *p* > 0.05). The mean age (SE) at which spectacle modalities were first recommended (bifocals or progressive lenses 9.5 (0.2) years, SV spectacles 9.2 (0.1) years, and myopia control spectacles 9.2 (0.2) years) was not statistically different from the mean age at which CL modalities were recommended (ortho-K 9.1 (0.2) years and myopia control CLs 8.8 (0.2) years; *p* > 0.05). The earliest intervention noted for CL modalities was 6.4 years for myopia control CLs and 6.5 years for soft multifocal CLs, demonstrating that optometrists are willing to fit CLs in younger children. In comparison, the earliest age at which spectacle modalities were recommended was 6.0 years for SV spectacles and 6.1 years for myopia control spectacles. Various atropine interventions—applied as monotherapy, in conjunction with myopia control, and in conjunction with SV spectacles —were implemented at similar ages of 8.5, 8.8 (0.9), and 8.3 (0.7) years, respectively.

#### 3.3.2. Patient Refractive Error

**Figure 3:**
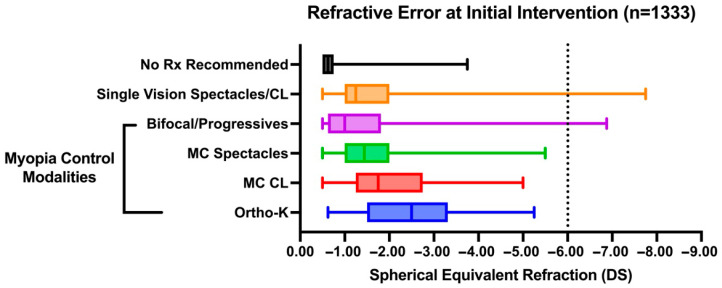
Box plot of patient refractive error across different modalities as first-line intervention recommended. Box boundaries denote upper and lower quartiles with the line within the box representing the median. Whiskers represent the full range of data points.

Myopia control CLs and ortho-K were overall prescribed for higher myopic refractive errors on average compared with SV spectacles and bifocals/progressives (*H*_5,892_ = 28.32, *p* < 0.0001). Myopia control CLs were prescribed at a significantly higher myopic refractive error (mean (SE) −1.78 (0.13) DS) compared with SV spectacles or SV CL (−1.33 (0.02) DS, *p* = 0.0049). Myopia control CLs and ortho-K (−1.93 (0.22) DS) were each prescribed at higher refractive errors than bifocals or progressive spectacle lenses (−1.23 (0.15), *p* = 0.0066 and *p* = 0.017), respectively. None of the patients with high myopia (greater than −6 DS, *n* = 25) were recommended a myopia control intervention as a first-line intervention (Figure 3). Overall, the results indicate that refractive error significantly influences the choice of initial management strategies.

**Figure 4:**
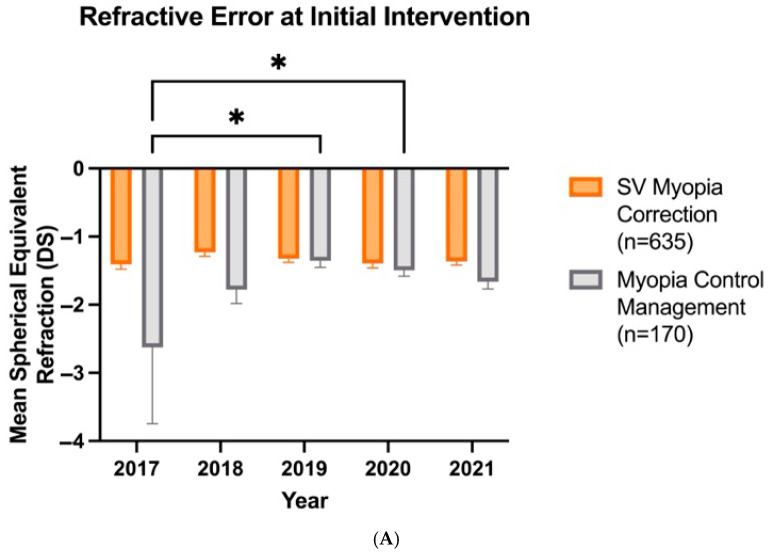

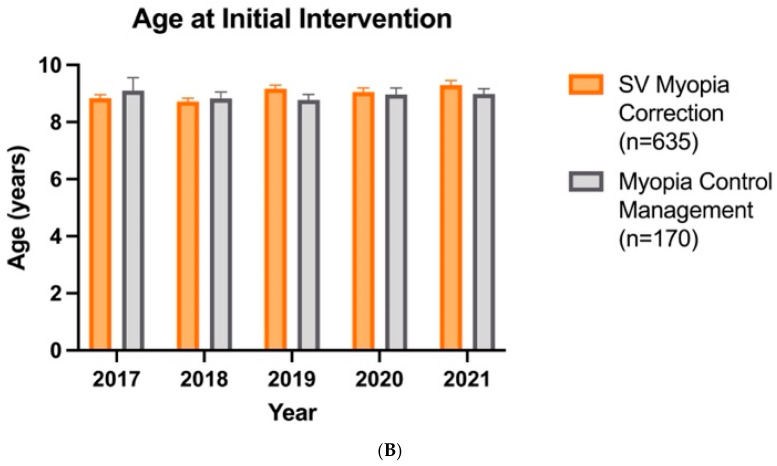
Patient refractive error (**A**) and age (**B**) at which SV myopia correction (*n* = 635) and myopia control management (*n* = 170) are prescribed as first-line intervention over the years. Error bars denote standard error, * denotes *p* < 0.05.

Over the years, there has been a shift in clinicians recommending myopia control at lower myopic refractive errors. The analysis of variance revealed a significant effect of treatment type (*F*_1,795_ = 20.08, *p* < 0.0001), year (*F*_4,795_ = 3.014, *p* = 0.018), and their interaction (*F*_4,795_ = 3.368, *p* = 0.0096). Compared with 2017, when clinicians recommended myopia control at a mean refractive error (SE) of −2.63 (1.12), myopic control recommendations were made at −1.35 (0.10) DS in 2019 and −1.49 (0.09) DS in 2020 (Figure 4A). However, the patient age at which myopia control is prescribed has not changed over the years (*F*_4,795_ = 0.85, *p* > 0.05), with a mean age of 8.93 (0.10) years (Figure 4B).

#### 3.3.3. Optometrist Year of Graduation

To determine if a practitioner’s year of graduation influenced the initial choice of myopia management, the median year of optometry school graduation for optometrists prescribing each intervention was analyzed. A Kruskal–Wallis test revealed that the initial intervention modality (choice) varies significantly with an optometrist’s year of graduation (*H*_5,1126_ = 22.23, *p* < 0.001). The median graduation year for optometrists who tended to prescribe bifocals and progressive spectacle lenses as initial intervention was 2003, compared with 2010 and 2012 for optometrists who initially prescribed myopia control spectacles and myopia control CLs, respectively.

### 3.4. Patient and Optometrist Factors That Affect the Prescribing of Myopia Control When Changing Management

There were 1302 visits (15.2% of all visits) where the optometrist altered the patient’s management (SV myopia correction or myopia control management, see Table 3). The VIF for all variables was less than 2, indicating a low level of multicollinearity that would inflate variance among explanatory factors. The model details can be found in Supplementary Materials Table S2. The logistic model revealed that significant predictors for the prescribing of myopia control management were: patient age at visit (*p* = 0.008), patient refractive error at visit (*p* < 0.001), parental myopia (one/both parents *p* < 0.001), optometrist’s graduation year (*p* = 0.003), and optometrist’s country of alma mater (*p* = 0.034). The likelihood of being prescribed myopia control increased 1.18 (95% CI 1.04–1.33) times per each additional year of age and 3.81 (95% CI 2.91–5.10) times per additional dioptre of myopia, with all other variables remaining constant. The odds of a clinician prescribing myopia control are 4.34 (95% CI 2.73–7.01) times greater if one or both parents are myopic compared with when neither parent is myopic. The likelihood of prescribing myopia control was 1.86 (95% 1.06–3.32) times greater if the optometrist was trained in Canada as compared with the United States. The odds of a graduate from 2010 prescribing myopia control were 1.46 (95% CI 1.14–1.88) times greater than those of a graduate from 2000.

## 4. Discussion

This retrospective chart review of optometry clinics in Ontario, Canada, revealed that, during the time period evaluated, while most clinicians continued to recommend SV myopia correction as the initial intervention for pediatric myopia, recommendations for myopia control increased over time. This increase coincides with the introduction of Health Canada-authorized commercially available myopia control soft CLs and spectacle lens options. Ontario optometrists are not alone in their reluctance to prescribe myopia control for their patients. A global survey of myopia management in 2016 found that the vast majority of practitioners prescribed single-vision interventions to young myopes, despite their awareness of myopia control options [24]. A more recent survey in 2022 found a greater uptake, with more practitioners prescribing myopia control interventions [20]. Despite the fact that the surveys were based on self-report and our study results were based on clinical data, the overall trends are similar. Furthermore, a retrospective chart review with an identical study design conducted concurrently in Hong Kong found similar increased adoption of myopia control over the same years studied (from 18.4% in 2017 to 42.8% in 2021) [25]. It should be noted that there remain significant disparities in access to evidence-based myopia control modalities worldwide.

Beyond revealing prescribing trends, this study design permits the quantitative analysis of factors that influence the prescribing of myopia control. These findings show that, although many optometrists in Ontario were waiting for evidence of myopia progression before introducing myopia control, the threshold of an “acceptable” amount of change has reduced by at least 1 D less myopia over the time period studied. Most of this change took place between the years of 2017–2020, when myopia control became available in daily disposable contact lenses in 2018 and spectacle modalities in 2019. Although myopia control was available in the form of ortho-keratology and atropine prior to this period, uptake may have been limited by the need for corneal topography equipment and a lack of commercially available atropine options. Thus, these interventions may have been considered only for when myopia was more severe. Published data from longitudinal studies may also have provided compelling evidence of the efficacy of myopia control. Through continuing education for clinicians and educational campaigns for the broader public, there is increasing recognition that myopia is known to progress, most rapidly in children before the age of 12, and there is no benefit in waiting for progression before initiating treatment [2]. Any myopia progression increases the patient’s risk of myopia-related ocular disease [8,19]. It is possible that proof of progression may be used as justification to both parents and optometrists that myopia control is required. Although Ontario optometrists are intervening with myopia control at lower levels of myopia over time, they appear to be hesitant to use myopia control in children presenting with higher levels of myopia. Published evidence is limited as to the efficacy of myopia control devices in patients with high myopia, as clinical studies have typically enrolled only patients with low to moderate myopia [26,27]. However, it is evident that any reduction in myopia progression is beneficial, and while there may not be published evidence for efficacy in patients with higher myopia, the possible benefits of myopia control may outweigh potential harms in this cohort [19].

The findings also reveal a preference for recommending spectacle modalities over contact lens forms of myopia control, which is corroborated by our Hong Kong companion paper [25]. In Ontario, the prescribing of myopia control spectacles surpassed that of myopia control CL beyond 2020. This may be attributed to the universal acceptance of and relative simplicity of fitting and wearing spectacle options, and possible concerns regarding the tolerability and safety of contact lenses and atropine. Clinicians and parents may have opted for myopia control without the perceived risk and side-effect profile of ortho-keratology, soft contact lenses, or atropine. Despite potential parental and patient concerns regarding pediatric contact lens wear, studies have shown that children and teenagers can wear contact lenses safely, with low rates of adverse events, similar to adults [28,29,30,31,32]. There are cosmetic and optical benefits to contact lens wear (particularly for patients with higher refractive errors), such as better vision-related quality of life, encouraging active lifestyles, and better compliance, as children are less likely to remove their contact lenses [33,34]. Clinicians were not hesitant to fit CLs even in the youngest children in our study sample.

A significant predictor for the prescribing of myopia control was awareness of a patient’s family history of parental myopia. This is particularly noteworthy given that a recent study found that children with myopic parents had faster-progressing myopia than those without parental myopia [35]. It is also likely that parents with myopia would be more receptive to the message and understand the importance of myopia control, recognizing the visual consequences of myopia firsthand. On the other hand, practitioners who had been in practice longer were more likely to prescribe older forms of myopia control (e.g., bifocals/progressives) than more recent graduates. While this may have been the most effective form of myopia control learned in school, newer forms of myopia control have been shown to have better efficacy. Similarly, optometrists trained in Canada were more likely to prescribe myopia control options compared with those trained in the United States. The reasons for this are unclear and this result may be impacted the imbalance of included practitioners who were trained in Canada as compared with the US and internationally. Overall, this reinforces the need for continuing education to keep clinicians up to date on the most effective treatments for myopia control and the availability of new modalities. Continuing education should provide guidance on how to initiate and modify treatments to best manage pediatric myopic patients. In its position statement on myopia management, the Canadian Association of Optometrists encourages optometrists to educate themselves and adopt evidence-based treatments to manage myopia progression [23].

On average, it takes 17 years for evidence to alter clinical practice patterns, and only 1 of 5 interventions become routine clinical practice [36]. Drawing from other healthcare disciplines, insights can be extracted on how to facilitate knowledge translation. Research has been conducted across healthcare disciplines to determine the factors involved in practitioner uptake of new devices and drugs. A systemic literature review of new drug uptake suggested that clinicians’ interest in particular therapeutic areas and participation in clinical trials increased the likelihood of early adoption [37]. Also noted was the importance of the marketing efforts of pharmaceutical companies and clinicians’ professional and social interactions. Changes in the management of diabetes and hypertension have also been studied. In 2023, Smith et al. stated that, for the provider, these barriers to change include cost, disrupted workflow, and the need to change office culture. Initiating myopia control management is a complex and time-consuming process that requires considerable staff time and both patient and parental interaction [38].

Notably, retrospective chart reviews do not reveal all the details of the discussions with patients and parents. It is possible that a discussion of myopia control occurred, but was not recorded. Since myopia control intervention may be more costly than single-vision correction, the discussion of myopia control intervention may be influenced by a patient’s socioeconomic status and private insurance coverage policies. Insurance coverage may vary across pharmaceuticals, spectacles, or contact lenses, which may influence parental choices. Also, these results may not be generalizable to other provinces because Ontario children have greater access to government-funded eye care than some other provinces and territories within Canada under their provincial health care plan. Practice patterns may vary in regions with limited access to local healthcare providers or restricted availability of certain management options (e.g., compounding pharmacies for atropine). Our findings underscore the importance of routine eyecare for children to ensure timely diagnosis of myopia and proactive management of children at risk of myopia. As with all chart reviews, not all parameters were available when not explicitly listed on patient charts (e.g., missing information on parental myopia and no information on patient ethnicity). Thus, the chosen logistic regression model included as many patient files as possible with the assumption that missing data were systematically distributed. While this approach was used to minimize systematic bias, it can result in wider confidence intervals for the odds ratio of some predictors where data are missing (such as for parental myopia). If missing information occurred in a biased way (e.g., information was not explicitly recorded moreso when neither parent was myopic), this may overestimate the likelihood that parental myopia is influencing prescribing decisions. However, our finding is corroborated by survey data finding that parental myopia does influence the decision to start myopia control, ranking after the consideration of patient age and refractive error [20]. Also, it was not possible to compute the change in refractive error in 51% of the cohort labeled as myopic at their first visit because they had no previous record. Recognizing that these are limitations inherent in chart reviews, these results may help to guide future randomized clinical trial design in identifying pertinent factors to consider. Furthermore, similar chart reviews should be conducted in other regions of the world to compare true practice patterns in order to examine cultural and socioeconomic influences on choice of myopia management interventions.

## 5. Conclusions

Although prescribing single-vision correction remains the predominant choice for the initial management of myopia through to 2023, optometrists in Ontario, Canada, are increasingly recommending myopia control options and becoming more proactive by intervening at lower levels of myopia. The findings from this study reveal evidence-to-practice gaps that should be the focus of continuing education programs. Clinicians should stay up to date about the most effective treatments for myopia control and integrate best evidence-based practices in the clinical management of myopic children.

## Figures and Tables

**Table 1:** Inclusion and exclusion criteria.

Category	Inclusion and Exclusion Parameters
Age	≥6 to ≤10 years old
Appointment time frame	≥1 appointment from 2017–2021
Spherical-equivalent refraction (SER)	Pre-myopia: ≥−0.25 to ≤+0.75 DS Myopia: ≤−0.50 DS
Cylinder	≥−1.00 DC
Binocular vision	No strabismus or amblyopia
Best-corrected visual acuity (BCVA)	≥20/30 in each eye
Other	No ocular or systemic disease, no developmental abnormalities

DS = dioptre sphere; DC = dioptre cylinder.

**Table 2:** Demographic characteristics of patient and optometrists included for data analysis.

Patients			
	Myopic Group	Pre-Myopic Patients Who Became Myopic	Total
N	1160	193	1353
Gender	598/1160 = 51.6% female	102/193 = 52.8% female	700/1353 = 51.7% female
Mean age at first visit (SD); Range	8.71 (1.37) years; 6.0–10.9 years	8.38 (1.43) years; 6.0–10.9 years	8.66 (1.38) years; 6.0–10.9 years
Spherical equivalent refraction (SER) at first visit (SD); Range	−1.44 (1.01) DS; −0.50 DS to −7.75 DS	−0.05 (0.27) DS; +0.75 to −0.38 DS	−1.25 (1.06) DS; +0.75 DS to −7.75 DS
Parental myopia	Neither 237/1160 = 20.4% One Parent 126/1160 = 10.9% Both Parents 68/1160 = 5.9% Unknown 729/1160 = 62.8%	Neither 37/193 = 19.2% One Parent 20/193 = 10.4% Both Parents 14/193 = 7.2% Unknown 122/193 = 63.2%	Neither 274/1353 = 20.3% One Parent 146/1353 = 10.8% Both Parents 82/1353 = 6.0% Unknown 851/1353 = 62.9%
**Optometrists**			
Gender	67 female (67/101 = 66.3%)		
Graduation Year Range	1977 to 2023		
Country of Alma Mater	Canada (72/101 = 71.3%) US (26/101 = 25.7%) International (3/101 = 3.0%)		

SE = standard error.

**Table 3:** Distribution of patient and optometrist factors considered for logistic regression.

Factor	Distribution
Management type	SV Myopia Correction *n* = 762 Myopia Control Management *n* = 540
Age at visit where management was changed	Mean (SD): 9.55 (1.77) years Range: 6 to 16.40 years
Rx at visit where management was changed	Mean (SD): −1.85 (1.23) DS Range: −0.50 to −11.375 DS
Parental myopia	Neither *n* = 242 One/both parents *n* = 263
Optometrist gender	Male *n* = 462 Female *n* = 840
Optometrist alma mater	Canada *n* = 913 US *n* = 389
Optometrist graduation year	Mean (SD): 2007 (10.26) Range: 1977 to 2023

SD = standard deviation.

## Data Availability

The dataset (comprised of clinical records) presented in this article is not readily available to preserve patient privacy. Access to the dataset may be granted upon reasonable request to the corresponding author, subject to institutional review and data sharing agreements.

## Supplementary Materials

The following supporting information can be downloaded at: https://www.mdpi.com/article/10.3390/jcm14145132/s1, Table S1: Breakdown of myopia management modalities for each patient (n=1160) at their initial encounter from 2017 to 2021. Table S2: Model summary (A), multicollinearity diagnostics (B) and coefficients (C) of binomial logistic regression.
