# Supplementary material for: Myopia Management in Ontario, Canada
Source: J Clin Med. 2025 Jul 19;14(14):5132. doi: 10.3390/jcm14145132 (PMC12295653; doi:10.3390/jcm14145132)
Supplement: Supplementary file 1 [file jcm-14-05132-s001.zip › jcm-3710176-supplementary.pdf]

Table S1. Breakdown of myopia management modalities for each patient (n=1160) at their initial encounter from 2017 to 2021.

	SV Spectacles/CL	No Rx Recommended	Myopia Control (Combined)	Myopia Control CL	Myopia Control Spectacles	Ortho-K	Bifocals/ Progressives	Atropine w SV	Atropine w MC	Soft Multifocal CL	Missing
2017	66.7%	27.7%	4.4%	0%	0.4%	2.8%	0.8%	0%	0%	0.4%	1.2%
2018	54.4%	32.0%	12.8%	8.0%	0%	2.4%	2.0%	0.4%	0%	0%	0.8%
2019	53.6%	30.8%	14.8%	7.6%	0%	2.5%	3.0%	0.4%	0.9%	0.4%	0.8%
2020	54.9%	27.2%	17.9%	3.5%	9.4%	1.5%	3.0%	0%	0.5%	0%	0%
2021	46.1%	23.1%	30.8%	3.2%	22.2%	1.8%	1.8%	0%	1.8%	0%	0%

Myopia Control (Combined) represents the sum of individual myopia control modalities (columns in grey).

Table S2. Model summary (A), multicollinearity diagnostics (B) and coefficients (C) of binomial logistic regression.

(A) Model Summary - Group

Model	Deviance	AIC	BIC	df	X^2	p
H_0	697.365	699.365	703.590	504		
H_1	476.672	490.672	520.244	498	220.693	< .001

(B) Multicollinearity Diagnostics

	VIF
Patient age at visit	1.032
Patient refractive error at visit	1.076
Parental Myopia (One/Both Parents)	1.100
Optometrist Gender	1.022
Optometrist Alma Mater	1.022
Optometrist Graduation Year	1.011

(C) Coefficients

					95 % Confidence Interval	
	**Estimate**	**Standard Error**	**z**	**p**	**Lower Bound**	**Upper Bound**
(Intercept)	-81.044	25.564	-3.170	0.002	-132.730	-32.196
Patient age at visit	0.164	0.062	2.639	0.008	0.043	0.287
Patient refractive error at visit	-1.337	0.142	-9.381	< .001	-1.629	-1.069
Parental Myopia (One/Both Parents)	1.467	0.239	6.126	< .001	1.006	1.947
Optometrist Gender - Female	0.078	0.249	0.313	0.754	-0.409	0.568
Optometrist Alma Mater - Canada	0.618	0.291	2.121	0.034	0.055	1.201
Optometrist Graduation Year	0.038	0.013	2.956	0.003	0.013	0.063

Note. Group level 'Myopia Control Management' coded as class 1.
